# Polyphenols as Adjuvant Treatment for Heart Failure with Preserved Ejection Fraction (HFpEF): A Review

**DOI:** 10.3390/antiox15030322

**Published:** 2026-03-04

**Authors:** Selma Guimarães Ferreira Medeiros, Rita de Cássia Avellaneda Guimarães, Aline Carla Inada, Carolina Di Pietro Fernandes, Rosângela dos Santos Ferreira, Karine de Cássia Freitas, Juliana Rodrigues Donadon, Valter Aragão do Nascimento, Priscila Aiko Hiane

**Affiliations:** 1 Graduate Program in Health and Development in the Central-West Region of Brazil, Medical School, Federal University of Mato Grosso do Sul, Campo Grande 79070-900, Brazil; draselmaguimaraes@gmail.com (S.G.F.M.); aline.inada@ufms.br (A.C.I.); rosangela.ferreira@ufms.br (R.d.S.F.); karine.freitas@ufms.br (K.d.C.F.); valter.aragao@ufms.br (V.A.d.N.); priscila.hiane@ufms.br (P.A.H.); 2 Pharmaceutical Science, Food and Nutrition Faculty, Federal University of Mato Grosso do Sul, Campo Grande 79070-900, Brazil; juliana.donadon@ufms.br

**Keywords:** heart failure with preserved ejection fraction, cardiometabolic dysfunction, oxidative stress and inflammation, bioactive compounds

## Abstract

Heart failure with preserved ejection fraction (HFpEF) is a complex clinical syndrome driven by systemic inflammation, persistent oxidative stress, endothelial dysfunction, and impaired mitochondrial bioenergetics. Despite recent therapeutic advances, the management of these specific pathophysiological mechanisms remains a challenge. Polyphenols, bioactive compounds found in plants, have emerged as potential modulators of these pathways. Objective: This review critically summarizes the pathophysiological and molecular evidence supporting the role of polyphenols—specifically phenolic acids, flavonoids, and lignans—in attenuating key pathways implicated in the progression of HFpEF, while also addressing the current limitations in clinical translation. Results: Preclinical evidence indicates that polyphenols regulate cellular homeostasis by activating the Keap1/Nrf2 antioxidant axis and AMPK/SIRT1 metabolic pathways, while inhibiting NF-κB-mediated pro-inflammatory signals and TGF-β fibrotic pathways. These molecular actions collectively preserve endothelial function via PI3K/Akt/eNOS, reduce interstitial fibrosis, and improve myocardial metabolic efficiency. Furthermore, the modulation of gut microbiota amplifies these systemic effects, particularly in obesity-related phenotypes. However, direct clinical application is currently hindered by low bioavailability and a scarcity of randomized trials specifically in HFpEF populations. Polyphenols represent a promising and biologically plausible nutritional therapeutic axis for the multidimensional management of HFpEF. While the molecular rationale is strong, future research should focus on improving bioavailability and conducting high-quality clinical trials to validate efficacy as an adjuvant therapy.

## 1. Introduction

Heart failure with preserved ejection fraction (HFpEF) represents a growing global challenge, accounting for over half of all heart failure diagnoses [1]. Characterized as a systemic syndrome, HFpEF is driven by comorbidity-induced microvascular inflammation and metabolic dysfunction, which lead to myocardial stiffness and interstitial fibrosis [2,3,4,5]. While Sodium-Glucose Cotransporter-2 (SGLT2) inhibitors have become the gold standard treatment, demonstrating significant reductions in hospitalizations, the heterogeneity of the disease necessitates adjunctive strategies targeting these residual inflammatory and oxidative drivers [6,7].

In this context, dietary polyphenols—including phenolic acids, flavonoids, and lignans—emerge as potent modulators of redox homeostasis [8,9]. By activating the Nrf2 pathway and inhibiting NF-κB signaling, these bioactive compounds help restore enzymatic balance, preserve mitochondrial integrity [10,11], and modulate gut microbiota-derived metabolites, thereby improving vascular stiffness and functional reserve [12,13].

Distinct from previous reviews that broadly address polyphenols in cardiovascular disease, this article focuses on the distinct pathophysiological mechanisms of the HFpEF phenotype. We integrate recent evidence on molecular targets—such as titin stiffness and microvascular inflammation—with the specific actions of polyphenol subclasses. Furthermore, we critically address the translational gap between preclinical findings and clinical application, identifying bioavailability and phenotypic heterogeneity as key hurdles to be overcome [14,15].

## 2. Heart Failure with Preserved Ejection Fraction (HFpEF): Concept and Epidemiological Overview

Heart Failure (HF) is characterized by the heart’s inability to meet tissue metabolic demands, manifesting as dyspnea, fatigue, and fluid retention. International guidelines establish specific clinical and echocardiographic criteria for diagnosis and stratification, reinforcing the contemporary understanding of the disease [16]. Advances in diagnostic methods and knowledge of cardiovascular physiology have enabled the consolidation of Ejection Fraction (EF) as an essential tool for the phenotypic characterization of HF. Because it is a multifactorial clinical syndrome, the disease has been classified into Heart Failure with Reduced Ejection Fraction (HFrEF, EF ≤ 40%) and Heart Failure with Preserved Ejection Fraction (HFpEF, EF ≥ 50%) [17,18], based on changes in titin, ventricular remodeling, and diastolic and systolic mechanical functions. HFpEF, also termed diastolic dysfunction, typically presents with heart failure symptoms, including dyspnea, exercise intolerance, and signs of congestion, even with a normal ejection fraction [19].

Structural and functional particularities characterize the pathophysiology that distinguishes HFpEF from HFrEF. While HFrEF involves contractile dysfunction, ventricular dilation, and exacerbated neurohumoral activation [19,20], HFpEF is characterized by concentric hypertrophy, increased myocardial stiffness, collagen accumulation, titin alterations, calcium handling dysfunction, atrial remodeling, and microvascular dysfunction [19,21,22].

These changes are exacerbated with aging and also when comorbidities such as systemic arterial hypertension, obesity, and type 2 diabetes mellitus are present, contributing to low therapeutic responsiveness, since effective interventions in HFrEF show limited benefit in HFpEF, reinforcing the need for individualized approaches based on clinical subphenotypes [23,24].

In recent decades, there has been an increase in the prevalence and incidence of HFpEF driven by population aging, increased survival of patients with HF, and a higher prevalence of chronic comorbidities such as systemic arterial hypertension, type 2 diabetes mellitus, obesity, and atrial fibrillation [25,26]. It is estimated that HFpEF accounts for a large proportion of heart failure hospitalizations in North America and Europe, with HFpEF comprising approximately half of all heart failure cases in contemporary cohorts [16,27]. Moreover, among the millions of Americans living with heart failure, a substantial share have preserved ejection fraction, reflecting the shifting epidemiology toward this phenotype [18,28]. The global prevalence of heart failure overall is estimated at 1% to 3% of the adult population, with prevalence rising sharply among older individuals aged 70 or older, a demographic in which HFpEF predominates [29,30].

The predominant clinical phenotype is women, elderly individuals, and those with multiple comorbidities, including chronic kidney disease, Chronic Obstructive Pulmonary Disease (COPD), anemia, and low-grade systemic inflammation, a profile that contributes to greater therapeutic complexity and a worse prognosis [31,32]. Cohort studies, such as ARIC and Framingham, reveal an even higher prevalence in populations characterized by socioeconomic and ethnic inequalities [22,33]. HFpEF accounts for between 28.2% and 59% of cases in outpatient services and between 20% and 53.1% in hospital settings, varying according to diagnostic criteria and care complexity [33,34], with hypertension and diabetes mellitus being the predominant comorbidities [35,36].

HFpEF presents lower mortality compared to HFrEF (5% to 8%); however, readmission rates are higher than 20% per year, reflecting its clinical complexity [7,35]. A recent study (REPORT-HF) indicates mortality rates of up to 22% in Latin America, in contrast to the 5% observed in the Asian registry ASIAN-HF [19,33]. Given this scenario, it is estimated that the costs related to heart failure reach USD 284 billion annually, with projections of doubling by 2030, mainly due to recurrent hospitalizations, the need for multidisciplinary follow-up, and reduced work productivity [28,30].

In this growing and multifactorial scenario of heart failure with preserved ejection fraction (HFpEF), early diagnosis and integrated treatment of comorbidities are essential for a better prognosis. Biomarkers such as NT-proBNP and troponins are fundamental tools for risk stratification and early identification of patients at risk of adverse outcomes [37].

However, interventions focused exclusively on pharmacotherapy have proven insufficient given the pathophysiological complexity of HFpEF, which is influenced by chronic low-grade inflammation, microvascular stiffness, endothelial dysfunction, and oxidative stress [19,38]. In this context, lifestyle change strategies, such as cardioprotective diets, regular physical exercise, and rigorous weight control, in addition to controlling visceral adiposity, emerge as tools with a significant impact on reducing hospitalizations and improving function [4,39].

Furthermore, health education programs, remote monitoring, structured dietary adherence, and integration across levels of care have demonstrated a significant impact on reducing readmissions and improving disease management, particularly among populations with greater socioeconomic vulnerability [38,40].

## 3. Clinical and Laboratory Diagnosis of HFpEF

The pathophysiology of HFpEF is complex, and there are no specific diagnostic markers. Furthermore, it is made more challenging by its association with other cardiorespiratory diseases, such as COPD, valvular heart disease, and physical deconditioning, in addition to the wide phenotypic heterogeneity of the syndrome [23,41]. HFpEF is defined by a Left Ventricular Ejection Fraction (LVEF) ≥ 50%, resulting from diastolic dysfunction and elevated ventricular filling pressures. Frequently, HFpEF coexists with comorbidities such as hypertension, visceral obesity, type 2 diabetes, atrial fibrillation, and chronic kidney disease, which contribute to worsening the prognosis and hindering diagnostic accuracy [11,42].

In this context, the diagnostic approach is systematic and integrative, consisting of a thorough clinical evaluation and the exclusion of differential diagnoses. Transthoracic echocardiography is the primary non-invasive method, providing fundamental data on LVEF, ventricular geometry, and diastolic function parameters such as the E/e′ ratio, septal and lateral e′ velocities, and the E/A ratio [2,41]. These echocardiographic markers enable estimation of ventricular filling pressures and guide classification of severity and associated risk.

Laboratory evaluation complements the diagnosis and assists in prognostic stratification. Measurement of natriuretic peptides, particularly *Brain Natriuretic Peptide* (BNP) and *N-terminal pro–Brain Natriuretic Peptide* (NT-proBNP), is widely recommended, with cutoffs of >125 pg/mL in an outpatient setting or >300 pg/mL in a hospital setting [3,23]. However, it is worth noting that normal values do not exclude HFpEF, especially in obese individuals, in whom the secretion of these peptides may be reduced. Even so, elevated NT-proBNP levels maintain substantial independent prognostic value, with a hazard ratio of 1.80 (95% CI: 1.38–2.35), reinforcing its role in the overall assessment [3,43].

Emerging biomarkers, such as proteomic signatures and circulating microRNAs, have shown promise in improving diagnostic accuracy by reflecting central mechanisms implicated in the pathophysiology of HFpEF, including inflammation, fibrosis, and oxidative stress [20,24]. These molecular markers have the potential to stratify patients into distinct clinical phenotypes, thereby enabling more personalized approaches.

When clinical, echocardiographic, and laboratory findings remain inconclusive, the use of validated diagnostic scores is recommended. The H2FPEF score integrates obesity, hypertension, atrial fibrillation, advanced age, elevated pulmonary systolic pressure, and increased E/e′ ratio, and enables objective classification of diagnostic probability, with excellent discriminative capacity [23,24]. In addition, the HFA-PEFF algorithm, proposed by the Heart Failure Association, organizes structural, functional, and laboratory parameters into specific domains, thereby providing a standardized diagnostic workflow that is widely adopted in international guidelines [44,45].

The investigation of associated comorbidities is part of the diagnosis. Laboratory tests, such as complete blood count, creatinine, electrolytes, Thyroid-Stimulating Hormone (TSH), ferritin, and C-Reactive Protein (CRP), are recommended to screen for frequently concomitant conditions, such as anemia, thyroid dysfunction, and chronic inflammation, all related to the worsening of functional limitations and the increase in symptomatology [15,44]. Evaluation of hormonal and inflammatory pathways also opens up complementary therapeutic perspectives, such as examining the effects of thyroid hormones on diastolic function [45] and using molecular panels to guide more precise interventions [15]. Thus, the structured integration of clinical evaluation, imaging, traditional and emerging biomarkers, validated diagnostic tools, and technological platforms enables improved characterization of HFpEF, thereby supporting individualized management and early interventions that improve prognosis and reduce complications associated with the syndrome.

## 4. Obesity and Its Contribution to the Pathophysiology and Progression of HFpEF

Obesity is one of the most prevalent factors in HFpEF, present in 80–90% of patients diagnosed with the syndrome [46]. Although Body Mass Index (BMI) represents the initial measure for classifying overweight (25.0–29.9 kg/m^2^) and obesity (≥30 kg/m^2^), its accuracy remains limited due to the heterogeneity of adipose tissue distribution. Current guidelines recommend complementing BMI with waist circumference, waist-to-height ratio, and body shape indices to improve stratification of cardiometabolic risk in HFpEF [47,48,49].

In addition to visceral obesity, excess subcutaneous adipose tissue contributes to increased peripheral vascular resistance and endothelial dysfunction, raising ventricular afterload and worsening diastolic dysfunction [48]. Perivascular Adipose Tissue (PVAT) accumulation promotes arterial stiffness and favors microvascular rarefaction, amplifying tissue ischemia and impairing myocardial perfusion [47]. Furthermore, the phenomenon of sarcopenic obesity, characterized by fat infiltration of skeletal muscle, reduces peripheral oxygen extraction capacity and exacerbates exercise intolerance, regardless of ejection fraction. Decreased muscle mass and increased intramuscular fat are associated with poorer functional performance in HFpEF [46].

Obesity is characterized by chronic low-grade inflammation, with elevated levels of pro-inflammatory cytokines, such as IL-6 (interleukin-6), TNF-α (tumor necrosis factor alpha), and leptin, which promote oxidative stress and activation of the renin–angiotensin–aldosterone system and the sympathetic nervous system. These mechanisms favor cardiac remodeling, myocardial fibrosis, and alterations in energy metabolism, contributing to the development of comorbidities such as type 2 diabetes, dyslipidemia, and sleep apnea in individuals with HFpEF [26,35,50]. Obese patients frequently present with low levels of BNP and NT-proBNP, which can delay the recognition of volume overload and compromise the accuracy of HFpEF diagnostic scores [23,26,50], demonstrating that obesity distorts the interpretation of classic biomarkers of heart failure.

In therapeutic management, the use of Sodium–Glucose Cotransporter 2 inhibitors (SGLT2i) in combination with Glucagon-Like Peptide-1 receptor agonists (GLP-1), such as semaglutide and tirzepatide, reduces visceral and systemic adiposity, improves the inflammatory profile, and attenuates the decline in diastolic function [7,51]. The American College of Cardiology recommends considering these drugs for obese patients with HFpEF, even in the absence of diabetes, given the observed benefits in quality of life [52]. In addition to SGLT2 inhibitors, mitochondrial metabolism modulators are promising strategies for HFpEF associated with obesity. The metabolic accelerator HU6 and selective adipose triglyceride lipase inhibitors (ATGL), such as atglistatin, promote specific reduction of visceral adiposity while preserving lean mass, resulting in hemodynamic improvements and greater functional capacity without compromising skeletal muscle [27,52,53,54].

**Figure 1:**
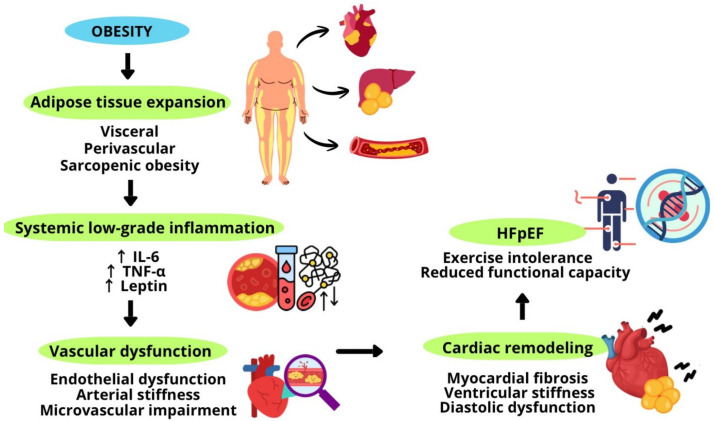
Obesity and its contribution to the pathophysiology of HFpEF.

In patients with morbid obesity and HFpEF, bariatric surgery maintains the most significant therapeutic impact, reversing ventricular remodeling, expanding functional capacity, and improving survival [55,56]. Furthermore, non-pharmacological strategies, including diets rich in antioxidant and anti-inflammatory compounds, resistance exercise, muscle reconditioning, and modulation of the gut microbiota, demonstrate synergy with pharmacological treatments, thereby enhancing metabolic and cardiovascular effects and contributing to the optimization of clinical outcomes [27,53,54]. Figure 1 summarizes obesity and its contribution to the pathophysiology of HFpEF.

## 5. Pharmacological Approaches and Adjunctive Therapies

The therapeutic challenges of HFpEF are evident, since there are still no pharmacological interventions capable of restoring or completely modifying the progression of the disease. Unlike HFrEF, which has therapies with proven efficacy in reducing mortality and hospitalizations, HFpEF lacks treatments with an equivalent impact [19,30,57].

Clinical management is based on rigorous control of associated comorbidities and optimization of intravascular volume through diuretics, in addition to heart rate control for symptomatic relief [58]. Among the most used therapies are SGLT2i, such as empagliflozin and dapagliflozin. Trials such as EMPEROR-Preserved and DELIVER have demonstrated a significant reduction in the combined risk of heart failure hospitalization and cardiovascular death in patients with an ejection fraction ≥ 40%, regardless of diabetes status [6,7,59].

Other pharmacological classes have also demonstrated benefits in specific subgroups of patients with HFpEF. Mineralocorticoid Receptor Antagonists (MRAs), such as spironolactone and finerenone, show particularly relevant benefits in individuals with chronic kidney disease and type 2 diabetes mellitus [60]. Complementarily, neprilysin inhibitors combined with Angiotensin II Receptor Blockers (ARNIs), such as sacubitril/valsartan, reduced hospitalizations in patients with borderline ejection fraction in the PARAGON-HF study, although without a significant impact on mortality [7]. In parallel, emerging therapies, including soluble guanylate cyclase stimulators such as vericiguat, GLP-1 receptor agonists, and glucagon receptor blockers, have the potential to reduce myocardial stiffness, particularly in inflammatory or metabolically compromised phenotypes [61,62].

Non-pharmacological approaches are consolidated as complementary strategies. Physical rehabilitation, with moderate-intensity aerobic training or combined with resistance exercise, promotes significant improvement in functional capacity and quality of life, even without substantial changes in echocardiographic parameters [25,63]. Caloric reduction and weight loss are also associated with improved diastolic function and reduced arterial stiffness in obese patients [31,32].

**Figure 2:**
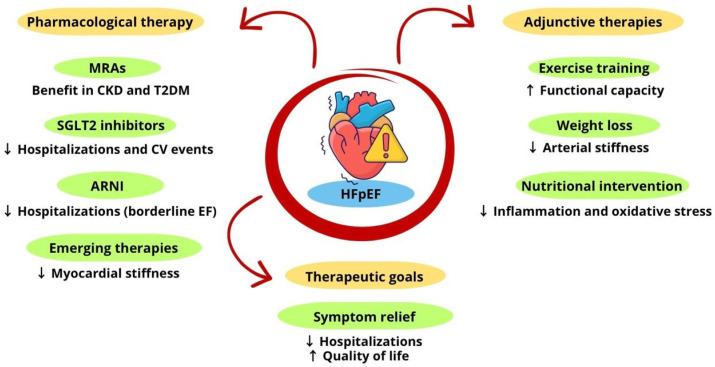
The principal pharmacological approaches and adjunctive therapies used in the treatment of HFpEF.

Nutritional intervention in HFpEF is an essential strategy, especially in the cardiometabolic phenotype characterized by visceral obesity, insulin resistance, low-grade chronic inflammation, and marked oxidative stress. Diets rich in antioxidant compounds and dietary fiber play a central role in modulating metabolic, hemodynamic, and immunoinflammatory pathways involved in diastolic dysfunction and ventricular stiffness, favoring, among other benefits, the reduction of body weight and the improvement of body composition, factors that directly influence the progression of the disease [44,47]. Figure 2 summarizes the principal pharmacological approaches and adjunctive therapies used in the treatment of HFpEF.

## 6. Nutritional Interventions and the Role of Antioxidant Compounds in HFpEF

Dietary interventions in HFpEF are based on dietary patterns rich in fiber, vegetables, fruits, and bioactive compounds, which modulate central pathways of inflammation, oxidative stress, and endothelial function. Dietary patterns such as the Mediterranean diet and the Dietary Approaches to Stop Hypertension (DASH) diet, both with a high density of antioxidants and bioactive compounds, encourage the consumption of foods rich in polyphenols and phytochemicals, such as extra virgin olive oil, which has a high phenolic content and exerts an antioxidant and vasoprotective effect with a direct impact on cardiac remodeling and endothelial function [12,64,65]. These compounds also modulate the gut microbiota, increase the production of Short-Chain Fatty Acids (SCFAs), and reduce systemic inflammation, being associated with reduced myocardial stiffness and improved microvascularization in HFpEF [12,66]. Therefore, dietary patterns rich in antioxidant compounds constitute a nutritional strategy that can attenuate the pathophysiology of HFpEF and provide complementary support for clinical management.

Dietary interventions can improve diastolic function, reduce arterial stiffness, and attenuate inflammation, possibly by increasing autophagy, modulating cellular senescence, and improving mitochondrial function [67]. When combined with physical exercise, caloric restriction potentiates the effects on functional capacity and quality of life [23,57]. Nutritional strategies with moderate carbohydrate restriction and a protein intake of 1.2–1.5 g/kg/day have been associated with reduced blood pressure, weight loss, improved diastolic function, and lower heart failure readmission rates, possibly mediated by attenuation of glucotoxicity and oxidative stress [68,69].

Vegetarian and pescatarian diets warrant attention due to their high intake of polyphenols and other bioactive compounds, which are associated with improved metabolic parameters, reduced inflammatory markers, and endothelial protection—mechanisms directly related to the pathophysiology of HFpEF [9,10,70].

There is a knowledge gap regarding clinical trials specifically using antioxidants or polyphenols in HFpEF. These compounds consistently affect central pathways, including modulation of oxidative stress, systemic inflammation, and endothelial dysfunction, underscoring their potential clinical utility as a complementary strategy in the management of HFpEF [68,69]. In this context, anti-inflammatory dietary patterns rich in antioxidant compounds emerge as tools to improve cardiac function, reduce hospitalizations, and contribute to a more favorable prognosis.

## 7. Polyphenols

Polyphenols, bioactive compounds found in plants, are considered important to metabolic health and are implicated in regulating weight, chronic disease, and cell proliferation [8]. Polyphenols can be consumed in the human diet through fruits and vegetables, as well as in polyphenol extracts used for supplementation or nutraceuticals [8].

Polyphenols constitute a class of bioactive compounds of interest for HFpEF due to their antioxidant, anti-inflammatory, and metabolic pathway-modulating properties crucial to the pathophysiology of the disease. Structurally, they contain one or more phenolic rings, which confer a high capacity to interact with molecular pathways that regulate oxidative stress and inflammation [57]. These are classified into subclasses, such as phenolic acids, flavonoids, stilbenes, and lignans, each with specific mechanisms that converge towards cardiovascular protection.

Despite their structural diversity, these compounds share core molecular mechanisms. They modulate redox homeostasis by reducing the production of Reactive Oxygen Species (ROS) and Reactive Nitrogen Species (RNS) and stimulating the Keap1/Nrf2 pathway. This activation promotes the transcription of endogenous antioxidant enzymes, including Superoxide Dismutase (SOD), Catalase (CAT), and Glutathione Peroxidase (GPx), which are essential for restoring cellular oxidative balance. Concomitantly, they inhibit NF-κB-mediated pro-inflammatory signaling, thereby reducing systemic cytokine and chemokine levels [9,10,70].

Furthermore, polyphenols play a significant role in preserving mitochondrial integrity, which is fundamental for myocardial bioenergetic efficiency and for preventing the worsening of diastolic dysfunction [56,62,71]. These compounds also modulate intracellular calcium homeostasis and activate cell-survival pathways, such as Silent Information Regulator 1 (SIRT1) and AMP-activated Protein Kinase (AMPK), thereby reducing apoptosis, lipotoxicity, and fibrotic remodeling [10,72]. These effects are especially relevant in patients with HFpEF, whose phenotype is characterized by chronic inflammation, microvascular dysfunction, and redox imbalance [55,67].

Finally, polyphenols exert a direct impact on endothelial function by inhibiting the oxidation of low-density lipoproteins (LDL-c), reducing cytotoxicity induced by oxidizing LDL-c, and halting the progression of vascular changes that contribute to cardiac remodeling [73]. Furthermore, they reduce Malondialdehyde (MDA) levels and promote positive feedback on endogenous enzymatic antioxidant systems, reinforcing the control of oxidative stress [10,58]. Thus, in addition to complementing traditional nutritional interventions, polyphenols represent an essential therapeutic axis in the multidisciplinary management of HFpEF, contributing to improved clinical outcomes, reduced hospitalizations, and greater long-term hemodynamic stability [10,12,74,75].

To provide a comprehensive overview of these molecular interactions, Table 1 summarizes both the shared signaling pathways—such as Nrf2 activation and NF-κB inhibition—and the distinct therapeutic targets specific to each polyphenol subclass, linking these targets to their respective physiological effects in HFpEF.

**Figure 3:**
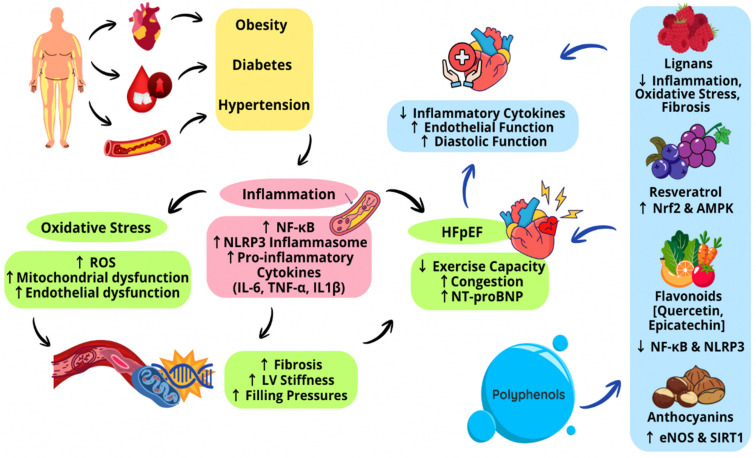
Comorbidities in HFpEF and the polyphenols that mimic inflammatory pathways.

Figure 3 below shows the integration of the different comorbidities acting in HFpEF and the different polyphenols mimicking inflammatory pathways.

### 7.1. Phenolic Acids

Phenolic acids are a subclass of metabolites that encompass approximately 8000 natural compounds. All phenolic acids possess a common structural characteristic, a phenol, which is an aromatic ring bearing at least one hydroxyl substituent [22,50,93]. They are widely distributed in fruits, whole grains, oilseeds, spices, coffee, and teas, and are divided into two main groups: benzoic acids and cinnamic acids [73,85]. These compounds modulate oxidative stress, inflammation, endothelial dysfunction, and energy metabolism. The principal phenolic acids that exert these effects and play central roles in the pathophysiology of HFpEF are gallic, ferulic, and chlorogenic.

#### 7.1.1. Gallic Acid

Gallic acid, found in grapes, strawberries, teas, and seeds, exerts antioxidant action and reduces pro-inflammatory pathways, specifically modulating Cyclooxygenase-2 (COX-2) and NF-κB signaling [74,77,94]. Beyond these general mechanisms, compounds with a structure similar to gallic acid act in the modulation of epigenetic pathways, reduce systemic inflammation, and influence cardiac remodeling, reinforcing its action in cardiometabolic diseases [68,78]. These findings are consistent with experimental models in which gallic acid reduces interstitial collagen, promotes ventricular hypertrophy reduction, and improves diastolic compliance [5,78].

#### 7.1.2. Ferulic Acid

Ferulic acid, present in whole grains, reinforces the Nrf2/ARE pathway, helping to activate antioxidant genes such as the Glutamate-Cysteine Ligase Catalytic Subunit (GCLC) and reduce vascular oxidative stress [65,76]. Furthermore, ferulic acid specifically inhibits Endothelin-1 (ET-1), thereby promoting improved insulin sensitivity and reducing inflammatory cytokines. Recent studies have shown that ferulic compounds interact with bioactive lipids and influence the lipid profile and systemic inflammation, strengthening their cardiometabolic role [64,77].

#### 7.1.3. Chlorogenic Acid

Chlorogenic acid, the primary polyphenol in coffee, reduces the activity of Nicotinamide Adenine Dinucleotide Phosphate (NADPH) oxidase, activates AMPK and Endothelial Nitric Oxide Synthase (eNOS), increasing NO production, and attenuates vascular inflammation [12,73]. Chlorogenic compounds also modulate the gut microbiota by increasing the production of SCFAs and reducing markers of cardiometabolic aging, which favors vascular function and energy metabolism [12,78]. These pathways are particularly relevant in HFpEF, which is characterized by chronic inflammation, arterial stiffness, and microvascular dysfunction. Thus, phenolic acids are biologically relevant molecules for the adjuvant treatment of HFpEF, contributing to improved diastolic function and hemodynamic stability [65,70].

### 7.2. Flavonoids

Flavonoids are an essential subgroup of dietary polyphenols, widely distributed in plant foods. They are recognized for their role in modulating antioxidant, inflammatory, and vascular pathways that are closely linked to the pathophysiology of HFpEF. Structurally, they have two aromatic rings connected by a heterocyclic ring [90]. Their dietary sources include red fruits, grapes, onions, green tea, cocoa, and wine, foods that account for a significant portion of dietary intake of these compounds [71].

Flavonoids primarily activate the Kelch-like ECH-associated protein 1/Nuclear factor erythroid 2-related factor 2 (Keap1/Nrf2) pathway, increasing the expression of HO-1, GCLC, SOD, CAT, and GPx, in addition to inhibiting NF-κB, reducing IL-6 and Tumor Necrosis Factor-alpha (TNF-α) [9,10,95,96]. These antioxidant mechanisms are particularly relevant in central alterations in the pathophysiology of HFpEF, which are characterized by cardiometabolic overload, systemic inflammation, and persistent oxidative stress [4,77,94].

The modulation of the Phosphoinositide 3-kinase (PI3K)/Akt/endothelial Nitric Oxide Synthase (eNOS), SIRT1, AMP-activated Protein Kinase (AMPK), and Peroxisome Proliferator-Activated Receptor Gamma Coactivator 1-alpha (PGC-1α) pathways contributes to increased Nitric Oxide (NO) bioavailability, improved mitochondrial function, and increased diastolic relaxation. These effects also align with the epigenetic action of dietary bioactive compounds, including Histone Deacetylase Inhibitors (HDACs), as discussed by Evans and Ferguson (2018) [68], which influence inflammatory pathways and cardiac remodeling relevant in HFpEF. Furthermore, the effects of flavonoids on lipid and carbohydrate metabolism significantly improve their regulation, thereby contributing to greater hemodynamic and metabolic stability [14,77]. These effects reduce the risk of adverse metabolic states common in heart failure, including the HFpEF phenotype, as demonstrated by Wang et al. (2023) [5].

#### 7.2.1. Quercetin

Quercetin, a flavanol widely present in foods such as onions, apples, and grapes, exhibits relevant antioxidant, anti-inflammatory, and antifibrotic effects. Beyond the shared Nrf2 and NF-κB modulation [41,79,80,81], quercetin specifically stimulates the AMPK pathway, favoring both fatty acid oxidation and glucose utilization. This metabolic modulation improves cardiac energy efficiency, reduces lipotoxicity, and contributes to myocardial protection, all of which are particularly important in HFpEF phenotypes associated with obesity and insulin resistance [10,24].

In the vascular endothelium, quercetin and other flavonoids increase NO bioavailability and decrease the expression of endothelial adhesion molecules such as Vascular Cell Adhesion Molecule-1 (VCAM-1) and Intercellular Adhesion Molecule-1 (ICAM-1), reducing leukocyte recruitment and vascular inflammation, as well as improving arterial compliance [81,83]. Regarding cardiac structural remodeling, quercetin inhibits signaling through the Transforming growth factor-β (TGF-β)/Smad pathway and reduces collagen synthesis and extracellular matrix accumulation, associated with the progression of HFpEF [10,82]. Rutin, a quercetin glycoside, further supports these effects by inhibiting the angiotensin-converting enzyme (ACE) and negatively regulating the Renin–Angiotensin–Aldosterone System (RAAS), helping to protect mitochondria against oxidative stress and reducing arterial stiffness [31,80,84]. Thus, by combining antioxidant, anti-inflammatory, metabolic, endothelial, and epigenetic effects, flavonoids act as prominent dietary agents in the management of HFpEF [65,90].

#### 7.2.2. Catechin and Epicatechin

Flavanols, especially catechins and epicatechin, constitute a subgroup of polyphenols widely present in cocoa, green tea, red wine, and infusions of *Camellia sinensis*. These compounds exhibit significant antioxidant, anti-inflammatory, and vasoprotective activities, pivotal in HFpEF given the presence of chronic low-grade inflammation and endothelial dysfunction [57,77].

Epicatechin is notable for its role in modulating cardiac energy metabolism via the SIRT1/PGC-1α and AMPK pathways, stimulating mitochondrial biogenesis, increasing fatty acid oxidation, and reducing lipotoxicity—a mechanism relevant to cardiometabolic phenotypes in pre-HFpEF [58,84]. In experimental models, epicatechin reduced myocardial fibrosis and oxidative stress, reinforcing its protective role on diastolic mechanics [71]. Furthermore, flavanols activate the PI3K/Akt/eNOS pathway, which increases NO synthesis, reduces arterial stiffness, and improves vascular compliance [81,85]. These vascular effects are associated with modulation of the gut microbiota and improved metabolic homeostasis [12,55].

Catechins, particularly epigallocatechin gallate (EGCG), exhibit potent activity by inhibiting NADPH oxidase and promoting eNOS phosphorylation [2]. EGCG also modulates antifibrotic pathways relevant to HFpEF remodeling, including attenuation of the Endothelial-Mesenchymal Transition (EndMT) and downregulation of the TGF-β/Smad pathway, reducing collagen deposition [3,25]. Its antifibrotic effects are characterized by strong convergence in the suppression of pro-inflammatory pathways, including COX-2 [68,72]. The combination of these effects makes flavanols dietary compounds of particular interest in supporting the treatment of HFpEF [10,87].

#### 7.2.3. Naringenin and Hesperetin

Flavanones, especially naringenin and hesperetin, present in citrus fruits, activate redox-protective pathways and promote improved insulin sensitivity and fatty acid oxidation via AMPK [78,87]. Additional modulation of the PI3K/Akt/eNOS pathway increases NO bioavailability and improves arterial compliance, fundamental to reducing the hemodynamic overload characteristic of HFpEF [88]. These mechanisms are critical given the correlation between insulin resistance and worse prognosis in heart failure [55], as well as the role of metabolic status and obesity in disease risk [39]. Furthermore, these dietary compounds modulate epigenetic processes and microbiota, which may amplify the protective effects of flavanones on vascular and metabolic function [12,68,87].

#### 7.2.4. Luteolin and Apigenin

Flavones, particularly luteolin and apigenin, found in parsley, celery, chamomile, and onion, exhibit specific anti-inflammatory and antifibrotic activity via modulation of the MAPK pathway (including p38 MAPK) and inhibition of the TGF-β/Smad cascade. This reduces fibroblast activation and extracellular matrix deposition, improving ventricular compliance [2,50]. Furthermore, these molecules increase Nrf2-regulated antioxidant gene expression and modulate mitochondrial bioenergetics, with potential implications for the functional determinants of HFpEF [4,53,65].

#### 7.2.5. Genistein and Daidzein

Isoflavones, particularly genistein and daidzein, found in soy and legumes, modulate endothelial function and glycolipid metabolism [52,89]. Beyond activating AMPK and Nrf2 [74,89,90], these compounds specifically interact with nuclear receptors such as Peroxisome Proliferator-Activated Receptor Gamma (PPARγ). This interaction promotes insulin sensitivity, reduces hepatic lipogenesis, and decreases the accumulation of intramyocardial triglycerides, effects of interest in HFpEF phenotypes associated with metabolic syndrome [55,71,77].

These effects promote greater NO bioavailability and improved diastolic relaxation [31,39,77]. Clinical trials in postmenopausal women have shown reductions in systolic blood pressure with genistein supplementation, although interindividual variability remains influenced by gut microbiota composition and the conversion of isoflavones into active metabolites [33,72,73]. In summary, the actions of flavanones, flavones, and isoflavones converge on central mechanisms of HFpEF, supporting the inclusion of polyphenol-rich diets as an adjuvant strategy [4,65,87,97].

#### 7.2.6. Anthocyanins: Cianidin, Delfinidin, and Malvidin

Anthocyanins are pigments characterized by a benzopyran nucleus, with cyanidin, delphinidin, and malvidin being the most prominent [64,77]. These compounds act on HFpEF by reducing ROS production through the inhibition of NADPH oxidase (NOX) and promoting AMPK activation [69,74]. They also increase eNOS activity, activating the NO–cGMP–PKG signaling cascade, fundamental to improving vascular compliance and diastolic function [81,84].

Cyanidin, found in blackberries and Brazilian açaí, stimulates eNOS phosphorylation and endothelium-dependent vasodilation while reducing TNF-α and IL-6 expression, suggesting relevant anti-inflammatory action for arterial stiffness [30,59,61]. Delphinidin, predominant in blueberries, has a high redox capacity associated with chelating action on metal ions (Fe^2+^ and Cu^2+^) and inhibition of endothelial adhesion molecules (ICAM-1 and VCAM-1) [41,60,91]. Malvidin, predominant in dark grapes, is notable for its chemical stability and modulation of the TGF-β1/Smad pathway. It also exerts an indirect effect via the gut microbiota, favoring the growth of Bifidobacterium and Lactobacillus, which contribute to endothelial function and metabolic homeostasis [87,91,98].

### 7.3. Lignans

Lignans are phenolic compounds derived from phenylpropanoid units, found primarily in seeds such as flaxseed, sesame, and sunflower, as well as in whole grains. Their action as complementary nutritional agents has demonstrated antioxidant, anti-inflammatory, and cardioprotective effects, with potential relevance in HFpEF [89,98].

The primary antioxidant mechanism of lignans combines direct neutralization of ROS with activation of intracellular pathways that regulate redox metabolism, particularly AMPK and SIRT1. SIRT1 activation leads to deacetylating and activating the coactivator PGC-1α, thereby stimulating mitochondrial biogenesis, increasing energy efficiency, and inducing the expression of endogenous antioxidant enzymes such as SOD, CAT, and GPx. Because of this, there is a reduction in oxidative stress and cellular damage, in addition to preserving the lipid, protein, and mitochondrial integrity of cardiomyocytes, and contributing to the maintenance of contractile function and ventricular compliance, with an essential effect on HFpEF [4,6,39].

In parallel, lignans inhibit the NF-κB transcription pathway, reducing the expression of pro-inflammatory cytokines such as TNF-α, IL-6, and IL-1β, conferring a significant anti-inflammatory effect. This modulation extends to the Transforming Growth Factor Beta 1/Mothers against decapentaplegic homolog 3 (TGF-β1/Smad3) pathway, in which lignans attenuate the activation of fibrogenic mediators, thereby inhibiting the transformation of fibroblasts into myofibroblasts and reducing the deposition of type I collagen.

Consequently, there is a reduction in interstitial fibrosis and preservation of myocardial architecture, critical mechanisms for limiting ventricular remodeling and maintaining diastolic function in patients with HFpEF. Furthermore, inhibition of this fibrogenic pathway reduces myocardial stiffness and hemodynamic overload, thereby integrating the anti-inflammatory and antifibrotic effects of lignans in cardiometabolic protection [14,17,32].

#### 7.3.1. Secoisolariciresinol-Diglucoside (SDG)

The most well-supported lignan is secoisolariciresinol diglucoside (SDG), whose primary dietary source is flaxseed. After ingestion, SDG is metabolized by the gut microbiota into active compounds, including enterodiol and enterolactone. These metabolites maintain systemic biological activity and activate SIRT1/AMPK pathways, thereby promoting mitochondrial biogenesis, oxidative phosphorylation, and cellular antioxidant defense.

Furthermore, increased expression of Nrf2 and antioxidant response element (ARE)-dependent genes, such as HO-1 and NQO-1, was observed, thereby strengthening cellular defense and mitochondrial integrity [70,77,92].

#### 7.3.2. Other Lignans: Matairesinol, Pinoresinol, and Lariciresinol

Other lignans, such as matairesinol, pinoresinol, and lariciresinol, exert complementary effects, including the modulation of apoptotic pathways (reduction of caspase-3 activation and mitochondrial cytochrome c release), decreased lipid peroxidation, and regulation of the extracellular matrix. These actions can attenuate pathological ventricular remodeling. Furthermore, lignans contribute to broader cardiometabolic benefits, such as improved lipid profile, increased insulin sensitivity, and positive modulation of the gut microbiota, promoting the generation of phenolic metabolites that amplify the reduction of systemic inflammation, favorably influencing the metabolic risk determinants for HFpEF [9,10,11,26].

The relevance of lignans in HFpEF stems from their association with obesity, insulin resistance, and heart failure risk. Subclinical metabolic alterations, common even in overweight individuals, can predispose to diastolic dysfunction and ventricular remodeling, scenarios in which lignan supplementation can act as an adjuvant modulator through antioxidant, anti-inflammatory, and metabolic effects [4,39].

**Figure 4:**
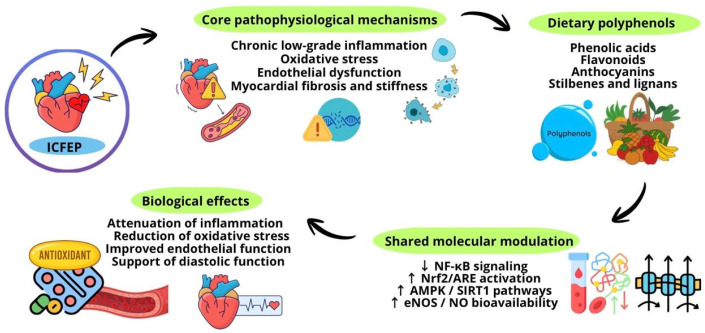
The role and contribution of polyphenols as adjunctive therapy in HFpEF.

Despite solid preclinical support, clinical data on HFpEF remain limited, underscoring the need for randomized, longitudinal studies that evaluate the impact of lignans in at-risk or diagnosed populations, using biomarkers of oxidative stress, endothelial function, fibrosis, and clinical outcomes [31,98]. Figure 4 summarizes the role and contribution of polyphenols as adjunctive therapy in HFpEF.

## 8. Clinical Evidence and Translational Challenges

Despite the robust preclinical evidence demonstrating the efficacy of polyphenols in modulating oxidative stress, inflammation, and myocardial stiffness, the translation of these findings to clinical practice in HFpEF remains a significant challenge. While epidemiological studies consistently associate polyphenol-rich diets (such as the Mediterranean and DASH diets) with reduced cardiovascular risk and improved diastolic function [12,65], randomized clinical trials specifically testing isolated polyphenols in HFpEF patients are scarce.

Most current clinical data are derived from HFrEF or general metabolic cohorts, leaving a gap in our understanding of how these compounds specifically affect the unique hemodynamics of the preserved ejection fraction phenotype. A major hurdle in this translation is the issue of bioavailability and dosing. Polyphenols generally exhibit poor systemic absorption and rapid metabolism. For instance, compounds like resveratrol and quercetin undergo extensive first-pass metabolism in the liver and gut, resulting in low plasma concentrations of the parent compound, which may differ significantly from the high doses often used in successful animal models [9,72]. Consequently, establishing an optimal therapeutic dose for humans remains difficult, as the linear dose–response relationship observed in vitro is rarely replicated in clinical settings.

### Human Clinical Evidence

While direct clinical trials investigating polyphenol supplementation specifically in patients with established HFpEF are limited, several randomized controlled trials (RCTs) have demonstrated the efficacy of these compounds in modulating key upstream pathophysiological drivers of the syndrome, such as hypertension, endothelial dysfunction, and early-stage hypertensive heart disease [99,100,101,102,103]. Specifically, studies involving curcumin and resveratrol have shown promise in reducing cardiac biomarkers like BNP and improving ventricular function [99,100]. Table 2 summarizes these representative human studies that illustrate the potential translational benefits of polyphenols on hemodynamic and inflammatory markers relevant to HFpEF.

Furthermore, interindividual variability plays a critical role. The therapeutic efficacy of polyphenols, particularly lignans and isoflavones, is heavily dependent on the host gut microbiota to convert precursors into active metabolites (e.g., equol from daidzein, enterolactone from lignans). Dysbiosis, which is common in HFpEF patients due to congestion and comorbidities, may therefore blunt the potential benefits of nutritional interventions [12,98]. Finally, given that HFpEF patients are typically elderly and subject to polypharmacy, future studies must also evaluate the potential pharmacokinetic interactions between high-dose polyphenol supplementation and standard heart failure therapies, such as SGLT2 inhibitors.

Studies have observed a higher risk of HFpEF in people with different metabolic health statuses who are overweight or obese. Obesity status is related as a cause in men (about 11%) and women (14%), and considering that body mass index (BMI) is the most widely used population marker for screening overweight and obesity in adults and older people, an increase of 1 kg/m^2^ increases the risk of HFpEF by 5% for men and 7% for women [27,104]. However, other adiposity measures are also used, such as waist circumference, which better identifies visceral adipose tissue [39]. Obstacles due to high measurement variability, including differences in anthropometric assessment conditions, the absence of variables for age, sex, and socioeconomic status, small sample sizes in studies, and a short follow-up period, limit comparisons with those reported in the literature.

Longitudinal randomized clinical trials are needed to monitor outcomes prospectively, considering the type of nutritional intervention, the use of nutraceuticals and nutritional supplements, and lifestyle changes, thereby identifying the sustained cardiac impact of body weight reduction, determining cause-and-effect relationships, and reducing bias.

Given the low oral bioavailability of polyphenols, valuable nutritional resources are being encapsulated in nanoparticles using bio-based polymers to form a protected, active complex with controlled release, which is biocompatible, biodegradable, and sustainable. As demonstrated by the phytochemical resveratrol, for example, dose-dependent, at a dosage of up to 5 g/day, it is considered safe in humans [105], improving cardiac function and remodeling, fibrosis, as well as metabolic regulation and lipid profile, increasing reendothelialization and reducing inflammation [106]. Efficacy has also been demonstrated with the administration of 0.5 mg/kg of curcumin using an oral drug delivery system formulated with a surfactant, resulting in a significant increase in gastrointestinal absorption [107].

Future directions for clinical research must prioritize: (I) the development of enhanced delivery systems (such as nanoparticle formulations) to improve stability and bioavailability; (II) the use of metabolomics to identify phenotypes based on gut microbiota composition; and (III) the design of long-term trials that evaluate hard clinical endpoints—such as hospitalization and exercise tolerance—rather than relying solely on surrogate biomarkers. Bridging this gap is essential to transform polyphenols from promising nutraceuticals into evidence-based adjuvant therapies for HFpEF.

## 9. Conclusions

The use of antioxidant compounds, especially polyphenols and their subgroups, such as flavonoids, anthocyanins, stilbenes, phenolic acids, and lignans, represents a promising and multifactorial strategy in the approach to HFpEF. Experimental evidence indicates that these compounds regulate key molecular pathways implicated in the pathophysiology of the disease, including oxidative stress, low-grade inflammation, endothelial dysfunction, and myocardial remodeling. The modulation of pathways such as Keap1/Nrf2, AMPK, SIRT1, and PI3K/Akt/eNOS reinforces the potential of these bioactive compounds to restore redox and mitochondrial homeostasis and to reduce fibrosis and improve diastolic function.

Despite this, the lack of studies relevant to clinical practice is a challenge, primarily due to variability in bioavailability and metabolism. Thus, future directions include developing formulations with greater stability and absorption, conducting specific randomized clinical trials in populations with HFpEF, and integrating personalized nutritional approaches based on the metabolic profiles of disease phenotypes. In this context, phenolic compounds are configured not only as therapeutic adjuvants but also as pillars of precision nutritional medicine aimed at modulating molecular mechanisms underlying HFpEF, with the potential to redefine preventive and therapeutic paradigms for this complex syndrome.

## Figures and Tables

**Table 1:** Summary of the main polyphenol subclasses, their representative compounds, and shared molecular targets implicated in the pathophysiology of Heart Failure with Preserved Ejection Fraction (HFpEF).

Polyphenol Subclass	Representative Compounds	Primary Molecular Targets & Mechanisms	Physiological Effects in HFpEF
Phenolic Acids	Gallic acid, Ferulic acid, Chlorogenic acid	↑ Nrf2/ARE: Upregulates antioxidant enzymes (SOD, CAT, GPx) [65,76]. ↓ NF-κB: Reduces pro-inflammatory cytokines [74,77]. ↓ ET-1: Inhibits Endothelin-1 signaling [65,76]. ↓ NADPH Oxidase: Reduces ROS generation [12,73].	Reduction of oxidative stress, improvement of endothelial function, and attenuation of systemic inflammation [12,65,78].
Flavonoids (Flavonols)	Quercetin, Rutin	↑ Nrf2 & ↓ NF-κB: Modulation of redox/inflammatory axis [41,79,80,81]. ↑ AMPK: Enhances energy metabolism [10,24]. ↓ TGF-β/Smad: Inhibits fibrotic signaling [10,82]. ↓ VCAM-1/ICAM-1: Reduces endothelial adhesion [81,83].	Attenuation of myocardial fibrosis, improvement of cardiac energy efficiency, and reduction of vascular inflammation [10,24,82].
	Catechin, Epicatechin, EGCG	↑ SIRT1/PGC-1α: Stimulates mitochondrial biogenesis [58,84]. ↑ PI3K/Akt/eNOS: Increases NO bioavailability [81,85]. ↓ NADPH Oxidase: Direct antioxidant effect [2]. ↓ EndMT: Attenuates endothelial-mesenchymal transition [3,25]	Improvement of diastolic relaxation, reduction of arterial stiffness, and preservation of mitochondrial integrity [55,65,86].
Flavonoids (Flavanones)	Naringenin, Hesperetin	↑ Nrf2 & ↓ NF-κB: Core antioxidant defense [78,87]. ↑ AMPK: Regulates fatty acid oxidation [78,87]. ↑ eNOS: Promotes vasodilation [88].	Improvement of insulin sensitivity and reduction of hemodynamic overload [39,55,88].
Flavonoids (Flavones)	Luteolin, Apigenin	↓ TGF-β/Smad: Strong antifibrotic action [2,50]. ↓ p38 MAPK: Reduces hypertrophic signaling [2,50]. ↑ Nrf2: Antioxidant gene expression [4,53].	Reduction of extracellular matrix deposition and improvement of ventricular compliance [2,50,65].
Flavonoids (Isoflavones)	Genistein, Daidzein	Agonist of PPARγ: Improves insulin sensitivity and lipid metabolism [55,71,77]. ↑ AMPK & Nrf2: Metabolic and redox regulation [74,89]. ↓ VCAM-1: Anti-inflammatory vascular effect [89,90].	Reduction of intramyocardial lipid accumulation and improvement of metabolic flexibility [55,77].
Flavonoids (Anthocyanins)	Cyanidin, Delphinidin, Malvidin	↓ NADPH Oxidase: Direct ROS scavenging [69,74]. ↑ NO–cGMP–PKG: Critical for cardiomyocyte relaxation [81,84]. ↓ TGF-β1: Antifibrotic effect [87,91].	Enhancement of vascular compliance and reduction of myocardial remodeling [30,81,84].
Lignans	SDG, Matairesinol, Lariciresinol	↑ SIRT1/AMPK: Mitochondrial and metabolic regulation [70,77,92]. ↓ NF-κB: Systemic anti-inflammatory effect [14,32]. ↓ TGF-β1/Smad3: Prevention of fibroblast activation [14,17].	Preservation of myocardial architecture and reduction of interstitial fibrosis [4,6,39].

**Table 2:** Summary of representative randomized clinical trials evaluating the effects of polyphenols on cardiovascular risk factors and mechanisms related to HFpEF pathophysiology.

Study/Reference	Population (n)	Intervention & Dose	Duration	Key Outcomes
Funamoto et al. (2022) [99]	Hypertensive Heart Disease (LVEF ≥ 60%) (n = 142)	High-absorption Curcumin (180 mg/day) vs. Placebo	24 weeks	Significant reduction in plasma BNP; no change in diastolic function (E/E’).
Militaru et al. (2013) [100]	Stable Angina/Heart Failure (Class I–II) (n = 40)	Resveratrol (20 mg/day) + Calcium vs. Control	60 days	Reduced NT-proBNP, total cholesterol, and inflammatory markers; improved systolic function.
Edwards et al. (2007) [101]	Pre-hypertensive and Hypertensive patients (n = 93)	Quercetin (730 mg/day) vs. Placebo	28 days	Reduced SBP (−7 mmHg) and DBP (−5 mmHg) in hypertensives; no effect in pre-hypertensives.
Barona et al. (2012) [102]	Metabolic Syndrome (n = 24)	Grape Polyphenols (46 g powder/day) vs. Placebo	30 days	Improved Flow-Mediated Dilation (FMD); reduced SBP and inflammatory cytokines (TNF-α).
Taubert et al. (2007) [103]	Pre-hypertension or Stage 1 Hypertension (n = 44)	Dark Chocolate (6.3 g/day, ~30 mg polyphenols) vs. White Chocolate	18 weeks	Improved endothelial function; reduced SBP (−2.9 mmHg) and DBP (−1.9 mmHg); increased NO bioavailability.

## Data Availability

No new data were created or analyzed in this study. Data sharing is not applicable to this article.
